# Quadrupeds of North America

**Published:** 1843-04

**Authors:** 


QUADRUPEDS OF NORTH AMERICA.

John James Audubon, Esq., the distinguished author of the magnificent work on the birds of North America, is now engaged, in conjunction with his two sons, upon a similar work respecting the natural history of the viviparous quadrupeds of the same region. This work is already considerably advanced, having been in progress for several years. It is to consist of thirty numbers, several of which are already completed. Mr. Audubon recently passed through our city, accompanied by several scientific gentlemen, on his way to the Oregon Territory, where he expects to spend several months prosecuting his researches. We had the pleasure of meeting him during his sojourn here. He was in fine health and spirits, his step as elastic as in youth, and his form still unbent and his eye undimmed by the suns and snows that have blanched his head. We were gratified with a view of some of the drawings for the forthcoming work, and were startled by the vivid and life-like portraits of some of the more familiar quadrupeds. His pencil seems to have lost nothing of its wonderful power, and we can assure the lovers of science every where, that a rich treat is in preparation for them, such as Audubon alone could give.

We cannot but look upon Mr. Audubon as one of the most remarkable men of the age. To our intellectual vision he stands solitary and alone. We are writing this paragraph almost in the midst of the scenes of his early labors, where, filled with the design of the great work which has made him world-renowned, he was struggling against mountain-difficulties that one of less bold and daring genius would have deemed insurmountable. Such they seemed to those around—and so utterly chimerical did his project appear that he had to encounter not merely the sneers and scoffs of enemies, but the lukewarmness and the estrangement of friends. After long years of toil, of labors to which the fabled ones of Hercules were but as girlish pastimes, when he had well-nigh completed the vast undertaking that has rendered his name immortal, he was still regarded as one whom long sojourn amidst the unbroken solitudes of nature and infrequent visits to the haunts of men had unsettled, and was considered a fit mark for rude gibes and jests. When at length he bore about him the splendid fruits of his martyr-like devotion, when the winged and beautiful inhabitants of our vast forests seemed flitting from spray to spray upon his canvass, or pouring forth their "wood-notes wild" along his enchanting page, obstacles seemed to thicken between him and the darling object of his life. Men heard and wondered—few believed. To publish was deemed the *ultima thule* of temerity. "My friends told me," said he, "to burn up my drawings and go home. I went abroad and the same advice was given me, "burn up your drawings and go home." But upborne by the divinity within, and true to its unerring instincts, he pressed forward, with the same iron will, the same indomitable energy, and lo! his name has gone abroad upon every wind—absolutely identified with the natural history of his native country!

Such a man does not appear in every age—it takes centuries to give birth to him. His whole history is full of interest. Not that it is singular; for it is the history of most great men. Such are seldom if ever appreciated in their early career by those immediately contemporaneous, which stamps them at once as the true nobility. Genius with its keen, sky-cleaving pinion ever holds its flight high above that of the multitude, and with far gaze pierces the future. C.

